# *Margaritaria nobilis* L.f. (Phyllanthaceae) Ethanolic Extract: Low Acute Oral Toxicity and Antinociceptive Activity

**DOI:** 10.3390/ph16050689

**Published:** 2023-05-03

**Authors:** Fabiana Menezes S. Camara, Brenda Costa da Conceição, Eloise Karoline S. Cardoso, Johan Carlos C. Santiago, Carlos Alberto B. Albuquerque, Washington L. Pereira, Marta C. Monteiro, Consuelo Y. Yoshioka e Silva, Milton Nascimento da Silva, Cristiane F. Maia, Eneas A. Fontes-Junior

**Affiliations:** 1 Laboratory of Inflammation and Behavioral Pharmacology (Lafico), Health Science Institute, Federal University of Pará, Belém 66075110, PA, Brazil; 2 Pharmaceutical Sciences Post-Graduation Program, Health Sciences Institute, Federal University of Pará, Belém 66075110, PA, Brazil; 3 Laboratory of Liquid Chromatography (Labcrol), Exact and Natural Sciences Institute, Federal University of Pará, Belém 66075110, PA, Brazil; 4 Animal Pathology Laboratory, Amazon Federal Rural University, Belém 66077830, PA, Brazil

**Keywords:** *Margaritaria nobilis*, natural products, medicinal plants, toxicity, nociception, pain, antinociceptive

## Abstract

*Margaritaria nobilis* L.f. (Phyllanthaceae), a native Brazilian tree occurring mainly in the Amazon, is used in folk medicine for the treatment of abscesses (bark) and cancer-like symptoms (leaves). The present study evaluates the safety of its acute oral administration and its effects on nociception and plasma leakage. The chemical constitution of the leaf’s ethanolic extract is determined by ultra-performance liquid chromatography–high-resolution mass spectrometry (LC-MS. Its acute oral toxicity is evaluated in female rats at a dose of 2000 mg/kg, evaluating the occurrence of deaths and Hippocratic, behavioral, hematological, biochemical, and histopathological changes, as well as food and water consumption and weight gain. Antinociceptive activity is evaluated in male mice with acetic-acid-induced peritonitis (APT) and formalin (FT) tests. An open field (OF) test is performed to verify possible interferences in the animals’ consciousness or locomotion. LC-MS analysis shows the presence of 44 compounds classified as phenolic acid derivatives, flavonoids and O-glycosylated derivatives, and hydrolyzable tannins. No deaths or significant behavioral, histological, or biochemical changes are observed in the toxicity assessment. In nociception tests, *M. nobilis* extract significantly reduces abdominal contortions in APT, demonstrating selectivity for inflammatory components (FT second phase), not interfering in neuropathic components (FT first phase) or consciousness and locomotion levels in OF. Additionally, *M. nobilis* extract inhibits plasma acetic-acid-induced leakage. These data demonstrate the low toxicity of *M. nobilis* ethanolic extract, as well as its effectiveness in modulating inflammatory nociception and plasma leakage, possibly related to the flavonoids and tannins present in its composition.

## 1. Introduction

Treatment of inflammation and pain management is still a major challenge for global public health. Although they are physiological processes, they are closely linked to the genesis and evolution of numerous pathologies [1]. Therefore, adequately treating inflammation and pain can positively impact patients’ clinical improvement and quality of life. This reality clarifies that analgesic and anti-inflammatory drugs are among the most prescribed and used in many countries, except for those where traditional medicine and medicinal plants are widely included in national public health policies [2].

Orthodox therapy, with steroidal (SAIDs) or non-steroidal (NSAIDs) anti-inflammatory drugs, opioids, antidepressants, antiepileptics, and sedatives, although efficient in many cases, find limitations in a significant variety of diseases, mainly when the risks outweigh the observed benefits [3]. In acute conditions, the need for high doses, which affect the stomach, liver, blood, and kidneys, is the main concern, in addition to respiratory and CNS depression, with possible cardiac changes as well [2,4]. Chronic conditions, however, which require prolonged or even continuous use of these medications, are the most challenging. In addition to worsening the adverse reactions already mentioned, they are associated with the induction of metabolic and immunological disorders, tolerance, dependence, and important behavioral impairments [3,4].

Therefore, the search for innovative therapeutic strategies, ranging from herbal medicines to new, safer, and more effective drugs, becomes imperative. In this sense, the medicinal culture of traditional populations proves to be an important source of information about plant species with pharmacological potential. It also provides clues about the safety of their use, reaffirming the value of ethnopharmacological knowledge [5]. 

Plant species *Margaritaria nobilis* L.f. (synonyms: *Phyllanthus antillanus* (A. Juss.) Müll. Arg.; *P. nobilis* (L.f.) Müll. Arg.; *P. ibonensis* Rusby; *Bradleia sinica* Müll. Arg.; *Cicca antillana* A.Juss.; *C. pavoniana* Baill.; *C. chinensis* Baill.; *C. surinamensis* Miq.; *C. sinica* Baill.; *Diasperus antillanus* (A. Juss.) Kuntze; *Margaritaria adelioides* Rich. ex Baill.; *M. alternifolia* L.; among others), popularly known as ‘botãozinho’ (little button), ‘figueirinha’ (little fig tree), ‘cabelo-de-cotia’ (agouti hair), ‘café-bravo’ (wild coffee) and ‘fruto-de-jacamin’ (jacamin fruit), for a long time was considered part of the genus Phyllanthus (family: Phyllanthaceae). Recently, however, it was reclassified based on phylogenetic studies, being now placed in the genus Margaritaria (family: Phyllanthaceae). This tree is native to Brazilian territory and distributed in all of the region’s states, emphasizing the Amazon [6]. In traditional medicine, its bark is cited for treating abscesses, fruits as a tonic, and leaves for treating cancer-like symptoms [7].

There are few studies regarding the chemical constitution of *M. nobilis*, such as the study by Moraes et al. [8], which addresses the phytochemical profile and leishmanicidal activity, and the study recently published with comprehensive characterization, indicating the presence of derivatives of phenolic acids, flavonoids, and hydrolyzable tannins [7]. In the genus *Margaritaria*, the presence of phenolic derivatives, glycosylated flavonoids (*M. discoidea*), and alkaloids (*M. indica*) has been described, together with their potential as antioxidant, antinociceptive, and anti-inflammatory agents [9,10,11,12,13]. The present study, therefore, aims to investigate the safety of acute oral administration of *M. nobilis* leaves ethanolic extract (MnE), considering behavioral, anatomical, histological, and biochemical aspects. It also seeks to elucidate its properties on nociception and inflammatory components.

## 2. Results

### 2.1. MnE Phytochemical Composition

**Figure 1:**
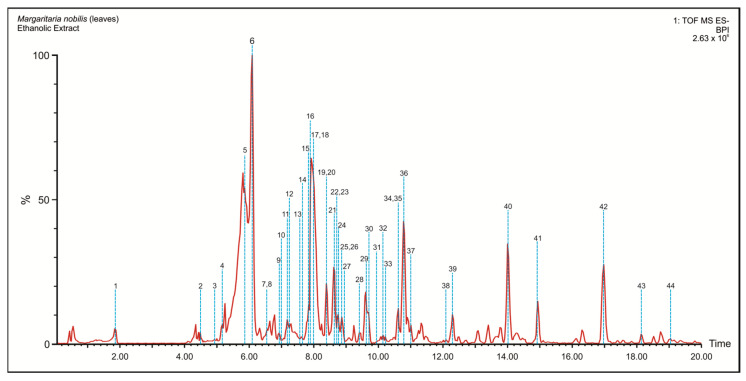
Total ion current chromatograms of *Margaritaria nobilis* ethanolic extract (MnE). The blue dashed lines indicate the retention time (RT) in which the compounds were recorded, being numbered in correspondence with Table 1.

Through the LC-MS technique, 44 chemical compounds of the sample under study were annotated. The chromatogram of total MnE ions can be seen in Figure 1; these compounds were classified into three major groups: phenolic acids, flavonoids, and hydrolyzable tannins, which can be seen in Table 1 with information on their *m/z* ratio, retention time, and their fragmentation patterns. Most of the annotated compounds are hydrolyzable tannins, a subclass of ellagitannins.

### 2.2. MnE Acute Oral Toxicity

#### 2.2.1. MnE Does Not Change Hippocratic Signs, Motor Behavior, or Emotionality, or Cause Deaths

**Figure 2:**
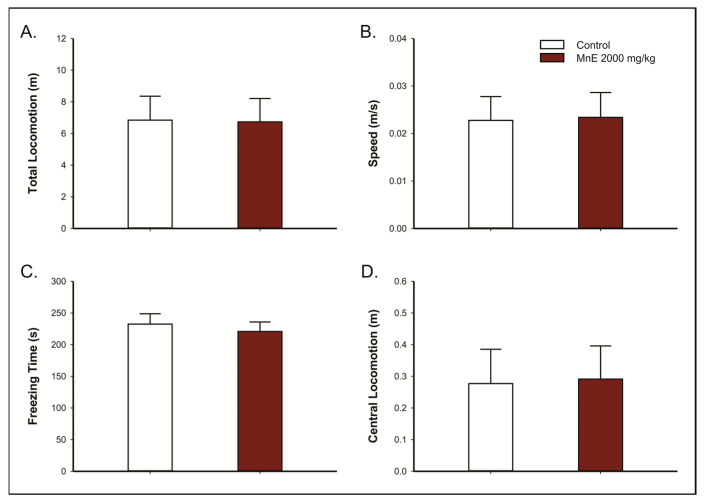
Effects of *Margaritaria nobilis* leaf’s ethanolic extract (MnE) acute oral administration (2000 mg/kg) on rats’ behavior in open field test, considering (**A**) total locomotion, (**B**) average locomotion speed, (**C**) freezing time, and (**D**) central locomotion. Data are presented as mean ± SEM (*n* = 5/group). Student’s *t*-test.

No significant changes in Hippocratic markers were observed in the four hours (15, 30, 60, and 240 min) following oral administration of MnE (2000 mg/kg), as well as in the 14 subsequent days. Similarly, oral administration of the extract did not alter motor parameters, such as total ambulation (6.72 ± 1.48 m; *p* = 0.960; Figure 2A) and locomotion speed (0.023 ± 0.005 m/s; *p* = 0.932; Figure 2B), or emotionality, such as freezing time (220.78 ± 14.93 s; *p* = 0.619; Figure 2C) and locomotion in the central area (0.29 ± 0.10 m; *p* = 0.928; Figure 2D) in the open field test, when compared to control animals (total locomotion = 6.84 ± 1.50 m; speed = 0.023 ± 0.005 m/s; freezing = 232.35 ± 16.39 s; central locomotion = 0.27 ± 0.11 m). No deaths were observed during the entire toxicity test period. 

#### 2.2.2. MnE Does Not Alter Water or Feed Consumption, Weight Gain, or Organ Histology

**Figure 3:**
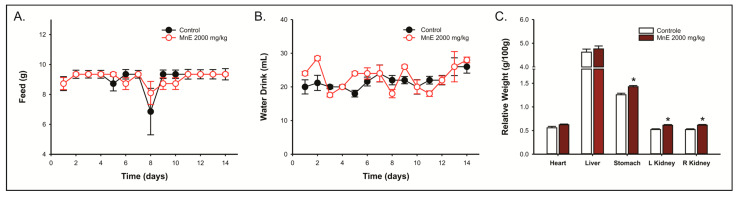
Effects of *Margaritaria nobilis* leaf’s ethanolic extract (MnE) on daily (**A**) feed and (**B**) water intake in the 14 days following the acute limit dose (2000 mg/kg) administration and (**C**) on the relative organ-body weights on the 14th day. Data expressed as mean ± SEM (*n* = 5/group). (**A**) Friedman test; (**B**) one-way RM-ANOVA; (**C**) Student *t*-test (* *p* < 0.05 vs. control group).

Animals treated with MnE (2000 mg/kg; v.o.) showed feed and water consumption patterns equivalent to those observed in the control group (Figure 3A,B), as well as weight gain. No changes in morphology, color, or size were observed during macroscopic analysis of the heart, liver, stomach, and kidneys. 

The stomach (1.427 ± 0.024 g/100 g b.w.; *p* = 0,00316) and kidney (L—0.610 ± 0.015 g/100 g b.w.; *p* = 0,00362; R—0.614 ± 0.015 g/100 g b.w.; *p* = 0,00303) relative weights of the animals treated with MnE were higher than the control group (stomach—1.256 ± 0.033 g/100 g b.w.; L kidney—0.521 ± 0.016 g/100 g b.w.; R kidney—0.519 ± 0.017 g/100 g b.w.). The heart and liver have relative weights equivalent to controls (Figure 3C). The histological evaluation did not reveal alterations indicative of toxicity, with histological normality being identified in all kidney and heart samples. Some animals (two controls and three treated) had mild chronic superficial gastritis not associated with acute treatment. The glandular and non-glandular regions of the stomach showed histological normality. Liver tissue, in general, was preserved, with histological normality. In some animals treated with MnE, the discreet presence of cytoplasmic vacuoles was observed but without repercussions for cell function (Figure S1).

#### 2.2.3. MnE Does Not Interfere with the WBC or Liver and Kidney Function Markers

**Figure 4:**
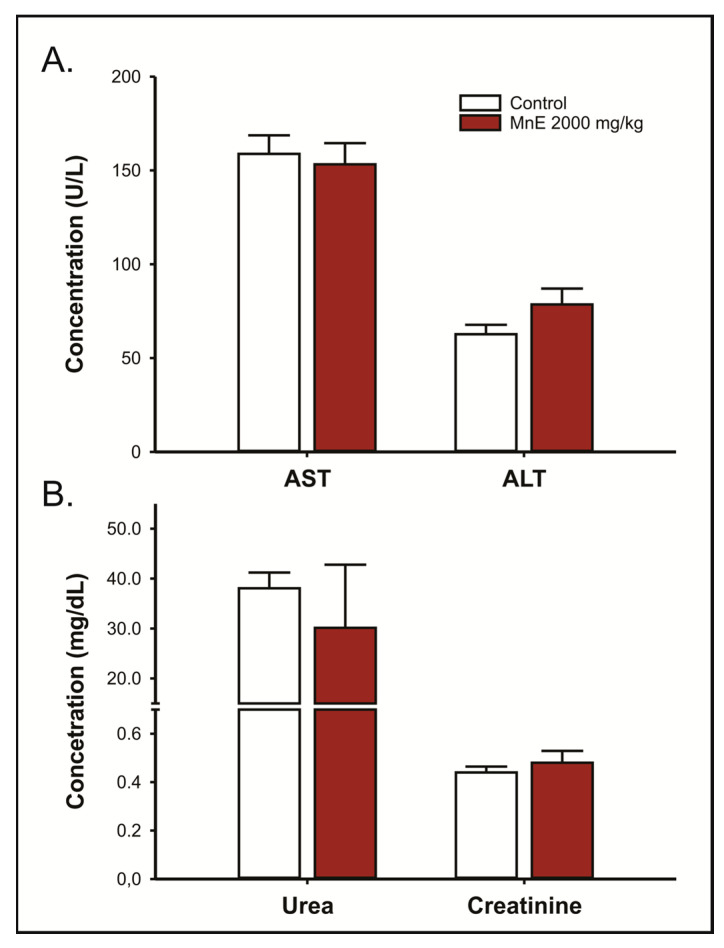
Effects of acute oral administration of *Margaritaria nobilis* ethanolic extract (MnE) (2000 mg/kg) on (**A**) alanine aminotransferase (ALT) and aspartate aminotransferase (AST) activity; and (**B**) urea and creatinine serum concentrations. Data expressed as mean ± SEM (*n* = 5/group). Student’s *t*-test.

MnE limit dose (2000 mg/kg; v.o.) administration did not change the leukogram of the animals (Table 2). Both groups had normocytic and normochromic red blood cells and no changes were observed in platelets. In addition, no changes were observed in the liver (AST and ALT; Figure 4A) and renal (urea and creatinine; Figure 4B) function markers, which showed plasma concentration equivalent to the control group.

### 2.3. MnE Antinociceptive and Antiinflamatory Activity

#### 2.3.1. MnE Reduces ACA-Induced Abdominal Writhing and Plasma Leakage

**Figure 5:**
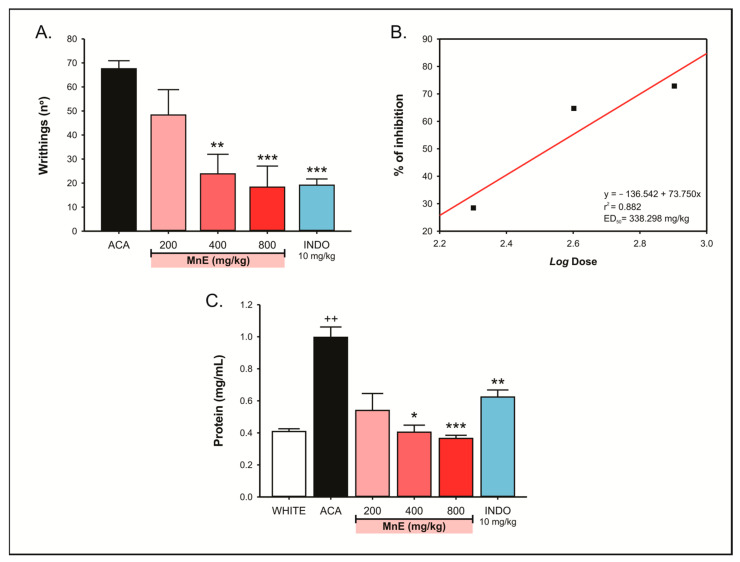
Effect of *Margaritaria nobilis* ethanolic extract (MnE) (200, 400, and 800 mg/kg; v.o.) on (**A**) acetic acid (0.6%)-induced abdominal writhing, with (**B**) identification of its median effective dose (ED_50_) by linear regression and (**C**) plasma leakage. Data expressed as mean ± SEM (A: *n* = 6–7/group; B: 5–6/group). ^++^ *p* < 0.01 vs. WHITE; * *p* < 0.05, ** *p* < 0.01, *** *p* < 0.001 vs. ACA; (ANOVA, Dunnett’s test).

Intraperitoneal administration of ACA (0.6%) produced 67.57 ± 3.33 writhes in the observation interval. The lowest MnE dose (200 mg/kg, v.o.), despite reducing the average of writhing (48.33 ± 10.54), did not present a statistically significant effect (*p* = 0.219). Doses of 400 and 800 mg/kg, on the other hand, reduced acetic-acid-induced writhing by 64.73% (23.83 ± 8.14; *p* = 0.001) and 72.87% (18.33 ± 8.76; *p* < 0.001), respectively, showing an effect like the indomethacin (10 mg/kg; 19.14 ± 2.56) (Figure 5A) and with an estimated median effective dose (ED_50_) of 338.298 mg/kg (r^2^ = 0.882) (Figure 5B).

Regarding proteins concentration in peritoneal fluid, the ACA (0.6%; ip.) administration increased it by around 144% (0.996 ± 0.065 mg/mL; *p* = 0.01) compared to the white group (0.408 ± 0.02 mg/mL), which did not receive ACA. Treatment with MnE at 200 mg/kg reduced the mean protein concentration (0.540 ± 0.105 mg/mL), but its effect was not statistically significant (*p* = 0.087) when compared to the ACA group. The dose of 400 mg/kg reversed the ACA-induced damage by 59.43% (0.404 ± 0.044 mg/mL; *p* = 0.003), showing an effect like indomethacin (10 mg/kg; 0.624 ± 0.044 mg/mL). At 800 mg/kg, MnE reduced the elevation in peritoneal fluid protein levels by 63.33% (0.365 ± 0.019 mg/mL; *p* < 0.001), showing an effect superior (*p* = 0.022) to that produced by the standard drug (Figure 5C). 

#### 2.3.2. MnE Reduces the Nociception in the Inflammatory Phase of the Formalin Test

**Figure 6:**
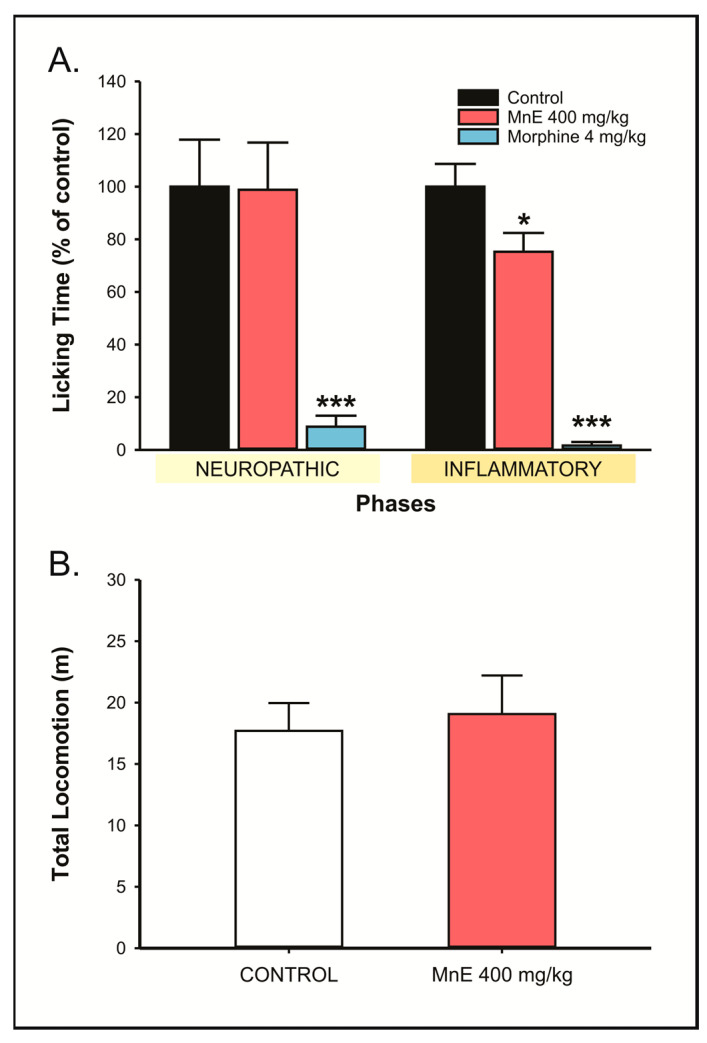
Effect of *Margaritaria nobilis* ethanolic extract (MnE) (200, 400, and 800 mg/kg; v.o.) on (**A**) neuropathic and inflammatory nociception in formalin test (**B**) and total locomotion in the open field. Data expressed as mean ± SEM (A: *n* = 6/group; B: *n* = 7/group). * *p* < 0.05, *** *p*< 0.001 vs. ACA; ((**A**) ANOVA, Dunnett’s test; (**B**) Student *t*-test).

Treatment with MnE (400 mg/kg; v.o.; 82.33 ± 14.96 s) did not interfere with formalin-induced licking time (83.33 ± 14.87 s; *p* = 0.998) during the neuropathic phase. In the inflammatory phase, on the other hand, MnE (400 mg/kg; v.o.; 129.17 ± 12.29 s) significantly reduced the licking time of the animals compared to the control group (171.67 ± 14.85 s; *p* = 0.031) (Figure 6A).

Additionally, the treatment with MnE (400 mg/kg; 19.06 ± 3.15 m) did not interfere with the locomotor capacity or exploration of the animals, when compared to the control group (17.69 ± 2.27 m; *p* = 0.809), excluding possible interferences in antinociceptive tests, such as sedation or motor impairment (Figure 6B).

## 3. Discussion

The present study demonstrates for the first time that MnE (ethanolic extract of *M. nobilis* leaves) has low toxicity when orally administered in an acute pattern, as well as its ability to modulate nociception, possibly by peripheral mechanisms related to inflammation, and to inhibit plasma leakage. Such effects are probably related to its secondary metabolites identified by our group, such as flavonoids, hydrolyzed tannins, and phenolic acids. We described the annotation of 44 phytoconstituents (Table 1) present in the MnE [7]. On this aspect, the metabolic annotation has expedited the clarification of the chemical-pharmacological signature of complex matrices, without ignoring the unequivocal identification that may allow bioactivity of greater magnitudes [14]. Thus, because some of these compounds are structural scaffolds for promising pharmacological segments, we contend that they may explain the observed activities interactively, such as gallic acid derivatives (1), quercetin O-glycosylated derivatives (40), and similar compounds (10, 14, 15, 21, 26, 28, 29, and 41), kaempferol (42) and its glycosylated derivatives, ellagitannins (analogs of 5–7, 17, 31, 38, and 48), and gallotannins (analogs of 8, 23, and 32).

However, research on the efficacy of natural products with therapeutic potential must be preceded by an investigation of the safety of their administration [15]. Secondary metabolites present in numerous genera are extremely important for their biological benefits, but their toxic capacity becomes a limiting factor for the use because of their risk [16]. In this sense, the present study is based on OECD guideline 425 and resolution nº 90 of the Brazilian health surveillance agency (ANVISA), which deals with the tests necessary for the registration of herbal medicines [17,18]. A limit dose (2000 mg/kg) of MnE was then administered orally, with subsequent evaluation of behavioral, physiological, anatomical, histological, and biochemical markers of toxicity.

Considering the lethality parameter, the limit dose administration did not cause immediate deaths over the 14 days of observation, suggesting the relative safety of the natural product. This observation is reinforced by the lack of physiological or behavioral changes that would indicate toxicity. Animals orally treated with MnE did not show changes in nutritional behavior or Hippocratic signs, nor in locomotion and emotionality parameters, evaluated in the open field. Such observations indicate that the extract, even in high doses, does not interfere with vital physiological functions, such as feeding, hydration, and weight gain, or alter the animals’ consciousness, mobility, and emotionality [19,20].

Even at therapeutic levels, several analgesic or anti-inflammatory drugs promote significant changes in the heart, stomach, liver, and/or kidneys, a feature that often limits the dose or duration of treatment [21,22]. The macroscopic and histological evaluation of these organs showed the absence of lesions when using the limit dose. These findings were reinforced by the absence of changes in biochemical markers, such as ALT, the most specific indicator of hepatocellular injury, AST, which can reveal lesions in the liver but also in other tissues, and urea and creatinine, markers of renal injury [15]. Similarly, the administration of the MnE limit dose did not promote hematological alterations. Given these findings and following OECD guidelines [17], MnE can be classified as a low-toxicity xenobiotic, with an estimated LD_50_ greater than 2000 mg/kg. Based on this classification, the evaluation of bioactive properties started with a dose of 200 mg/kg, corresponding to 10% of the dose tested in the toxicity assay, being adjusted based on the response, also testing doses of 400 and 800 mg/kg.

Initially, the MnE antinociceptive property was evaluated in the acetic-acid-induced peritonitis model, a classic test with high sensitivity, useful for screening drugs with antinociceptive potential. Intraperitoneal administration of acetic acid triggers local nonselective activation of cation channels, peritoneal membrane irritation, mast cells, and macrophages activation, the elevation of proinflammatory enzyme (nitric oxide synthase, cyclooxygenase, etc.) and mediator (cytokines, prostanoids, leukotrienes, bradykinin, serotonin, nitric oxide, etc.) levels [23]. The installed inflammatory process activates visceral nociceptors, causing primary hyperalgesia and intense nociception, manifested in the form of abdominal writhing [24,25]. Due to its characteristics, acetic-acid-induced peritonitis is sensitive to drugs capable to modulate inflammatory mechanisms of nociception, such as NSAIDs, although its nociception is also inhibited by other types of agents, such as opioids and antispasmodics [23,26]. Our trials demonstrated that MnE, at doses of 400 and 800 mg/kg, reduced by ~65% and ~73%, respectively, the acetic-acid-induced inflammatory nociception, with an estimated ED_50_ of 338.298 mg/kg, suggesting a potential for inflammatory pathways modulation, especially prostaglandins and nitric oxide.

A second consequence of acetic-acid-induced acute peritonitis is vasodilatation and increased vascular permeability, related to mediators such as histamine, prostaglandins, and nitric oxide. This condition favors the migration of leukocytes and plasma proteins to the peritoneal cavity [27]. In fact, in our tests, a great increase (~144%) in protein concentration was observed in the peritoneal fluid of animals administered with acetic acid. Treatment with MnE, mainly at doses of 400 and 800 mg/kg, completely inhibited plasma extravasation, promoting peritoneal protein concentrations equivalent to those of animals that were not injected with acetic acid (white group), reinforcing its ability to modulate inflammatory mechanisms such as vascular relaxation, diapedesis, and plasma leakage, which is associated with the elevation of interleukins, prostaglandins, and nitric oxide, potential targets of MnE action [28].

Next, the formalin test was carried out to evaluate MnE influences on peripheral and central components of nociception, since the intraplantar injection of formalin (2.5%) induces a characteristic biphasic nociceptive process. In the first 5 min, the nociception observed is due to direct stimulation of sensory terminals, corresponding to the neuropathic component of the test. In this phase, the modulation of nociception has been linked mainly to drugs capable of interfering with neural mechanisms that conduct the noxious stimulus, such as opioids, anticonvulsants, and antidepressants, among others [23,27,29]. MnE did not promote alterations in the neuropathic component of formalin-induced nociception, indicating that its constituent probably does not act on nervous components. A 10-minute hiatus is observed in the formalin test, which is followed by a new period (15–30 min) of nociceptive manifestation, now caused by an inflammatory process triggered by formalin aggression, which involves prostaglandins, histamine, serotonin, and NO and plasma leakage [29,30]. MnE (400 mg/kg) significantly reduced formalin-induced inflammatory nociception. 

Some of the secondary metabolites annotated by our group in the sample under study, such as ellagic acid, *p*-coumaric acid, and quercetin 3-O-xyloside, have been attributed to antioxidant activity, with the ability to scavenge reactive oxygen species. Ellagic acid is also capable of positively modulating Nrf2, a key mediator in the regulation of oxidative balance [30,31,32,33]. Oxidative stress and inflammation are closely related, as the overproduction of reactive oxygen and nitrogen species intrinsically participates in the cellular and humoral response, also influencing the inflammatory mechanisms of pain [30]. Furthermore, ellagic acid, gallic acid, methyl gallate, *p*-coumaric acid, quercetin 3-O-xyloside, quercetin, and kaempferol have also demonstrated important anti-inflammatory activity, related to the suppression of synthesis and release of pro-inflammatory cytokines (e.g., IL-1β, IL-6, IL-17, TNFα, INFγ) and elevation of anti-inflammatory cytokines (e.g., IL-4, IL-10) and to the reduction in expression and activity of pro-inflammatory enzymes (e.g., COX-2, iNOS) and mediators (e.g., PGE2, NO), in addition to the ability to modulate nuclear factor kB (NF-kB), a key element in the inflammatory response regulation, attributed to *p*-coumaric acid [30,32,33,34,35,36,37,38,39,40].

Such observations, in association with our findings, reinforce the thesis that the reduction in acetic-acid- and formalin-induced nociception and acetic-acid-induced leakage promised by MnE is due to the modulation of pain inflammatory components, possibly a product of the synergistic action of its metabolites. We cannot, however, rule out other accessory mechanisms, such as antihistamine, antispasmodic or adrenergic activity, which should be explored in future essays. The other compounds annotated by our group, which lack studies on their biological properties, should also be explored. The present study, by exposing the phytochemical profile of MnE, annotating 44 constituent substances, and demonstrating its low acute toxicity and antinociceptive potential, paves the way for new studies on the medicinal use of *M. nobilis*, expanding knowledge about the safety of its use, considering repeated administrations in a subchronic or chronic pattern, its antioxidant and anti-inflammatory properties, and underlying mechanisms—both of its extracts and the molecules identified.

## 4. Materials and Methods

### 4.1. Chemicals and Reagents

In the present study, all reagents were of analytical grade. As an extraction solvent, 99% absolute ethanol was used (Exodus Scientific, São Paulo, SP, Brazil). Ultrapure water, acetonitrile, and concentrated formic acid solution, obtained from Merck (Darmstadt, Germany), were used to prepare the mobile phase and solubilize the sample for LC-MS analyses. The dimethyl sulfoxide (DMSO, Sigma-Aldrich, St. Louis, MO, USA), ALT, AST, urea, and creatinine assay kits was from VIDA (Belo Horizonte, MG, Brazil). The acetic acid was from (ACA) and formaldehyde from (Vetec Química Fina, Rio de Janeiro, RJ, Brazil), the indomethacin from (Sigma-Aldrich, St. Louis, MO, USA) and morphine sulfate from (Cristália, Rio de Janeiro, RJ, Brazil).

### 4.2. Plant Collection and Extract Preparation Protocol

Leaf samples of *M. nobilis* (~1 kg) were collected at 1°02′08″ S and 46°49′41″ W (forest region of Bragança-PA, Brazil), identified by botanist Nascimento, E.A.P. (Brazilian Company of Agricultural Research—EMBRAPA), and a voucher specimen was deposited in its IAN herbarium (registration nº 191496).

Botanical material was washed, dried in a circulating oven (45 °C) until constant weight, and then pulverized in a ball mill (Fritsch, Idar-Oberstein, Germany). The semi-fine powder (60–100 µm) obtained was subjected to two sequential 24 h cycles of extraction with ethanol (99%; 4 L for each 1.0 kg of powder). The extract was concentrated in a rotary evaporator (Büchi, Flawil, Germany) and dried in an oven (40 °C) until constant weight.

### 4.3. Phytochemical Analysis

Metabolic characterization of MnE was obtained using a liquid chromatography system coupled to a time-of-flight mass spectrometer and triple quadrupole analyzer (model UPLC-QTf Xevo G2-S™, Waters, Milford, MA, USA). The chromatographic separation method was a 30 min gradient elution (0–2 min, 10–20% B; 2–30 min, 20–50% B). A 2 µL aliquot of the extract (3 mg.mL^−1^) was eluted on a BEH C18 column (Waters, Wexford, Ireland; 50 × 2.1 mm i.d., particle size 1.7 μm) at 40 °C and under a flow of 0.3 mL/mL of ultrapure water (solvent A) and acetonitrile (solvent B) + 0.1% (*v*/*v*) formic acid. The analyzed ionization mode was negative with a mass range of 100 to 1200 *m/z* and a scanning interval of 0.1 s. The source was maintained at 120 °C and the gas flow was adjusted to 50 L.h^−1^. The desolvation gas was set to 800 L.h^−1^ and 450 °C. The capillary voltage was set at 2.0 kV with cone voltage at 80 V. Data acquisition and processing was performed by MassLynx software licensed from Waters^®^.

### 4.4. Animals

Female Wistar rats (150–200 g) and male mice (25–30 g) were provided by the Federal University of Pará (UFPA) vivarium. They were kept in a standardized environment, with exhaustion, acclimatization (22 ± 1 °C) and light cycle (light 6 a.m. to 6 p.m.), in polypropylene cages (39 × 32 × 16 cm; up to five/box). Water and food were available ad libitum [39]. The experimental protocols were approved by the UFPA ethics committee (protocol nº 9568260617) and performed in a sound-attenuated laboratory under low-intensity light (12 lux), between 12:00 a.m. and 5:00 p.m.

### 4.5. Drug Solutions and Administration

MnE was solubilized in saline solution added with 4% DMSO. Standard drugs were dissolved in 0.9% saline. ACA and formaldehyde were dissolved in distilled water. Solutions were prepared with an administration pattern according to body weight (mice: 0.1 mL/10 g; rats: 0.1 mL/100 g). The oral (v.o.; gavage—MnE and indomethacin), subcutaneous (sc.—morphine), intraperitoneal (ip.—ACA), and intraplantar (formaldehyde) routes were adopted. 

### 4.6. Acute Oral Toxicity

#### 4.6.1. Treatment and Hippocratic Screening

The toxicity study was conducted by Guidelines for Testing Chemicals nº 425 of the Organization for Economic Cooperation and Development (OECD) and resolution 90 of the Brazilian health surveillance agency [18,41]. Therefore, female rats (five/group) were treated with 0.9% saline added with 4% DMSO (control) or a limit dose (2000 mg/kg) of MnE.

Manifestation of signs of toxicity and death was then evaluated, as described by Malone (1977) [42], at intervals of 0, 15, 30, 60, and 240 min after administration, and daily thereafter for 14 days. Animals’ weights, as well as the consumption of water and feed, were recorded daily too. The rats were euthanized by cervical dislocation under anesthesia at the end of the evaluation period. Blood samples were then collected by a ventricular puncture for hematological and biochemical assays. Heart, liver, stomach, and kidneys were collected for relative weight and macroscopic and histological characteristics evaluations.

#### 4.6.2. Open Field Test (OF)

The open field test was performed 4 h after MnE limit dose (2000 mg/kg) administration to evaluate possible harmful effects on mobility and emotionality. For that, animals were positioned individually in the center of a square arena (width 100 × depth 100 × height 40 cm), the spontaneous locomotion being evaluated for 5 min [19]. The experiment was recorded by a cam positioned above the arena. Total locomotion, locomotion speed, freezing time, and locomotion in the center of the arena were evaluated using ANY-maze^®^ (Stoelting Co., Wood Dale, IL, USA) software.

#### 4.6.3. Blood Count

Differential leukocyte counts and evaluation of possible abnormalities in red blood cells, leukocytes, and platelets were performed using the blood smear technique. Therefore, smears were prepared immediately after blood collection by the wedge method [43]. The evaluation was performed using an optical microscope (Nikon Eclipse E200, Melville, NY, USA).

#### 4.6.4. Biochemical Assays

##### Sample

Blood samples were subjected to centrifugation (10 min at 1400× *g*) for plasma separation, which was used for alanine aminotransferase (ALT) and aspartate aminotransferase (AST) activity determination and urea and creatinine concentration.

##### Hepatic Function Assays

Alanine Aminotransferase (ALT) Activity

Determined through the reaction between pyruvate, formed by the transfer of amino groups from alanine to ketoglutarate, catalyzed by ALT, and NADH, forming L-lactate and NAD+, catalyzed by lactate dehydrogenase (LDH). ALT catalytic concentration (U/L) was determined from the rate of consumption of NADH, measured by spectrophotometry (λ = 340 nm) [44,45].

Aspartate Aminotransferase (AST) Activity

Determined through the reaction between oxaloacetate, formed by the transfer of amino groups from aspartate to ketoglutarate, catalyzed by AST and NADH, forming L-malate and NAD+, catalyzed by malate dehydrogenase (MDH). AST catalytic concentration (U/L) was determined from the rate of NADH consumption, measured by spectrophotometry (λ = 340 nm) [45,46].

##### Kidney Function Assays

Creatinine

Serum creatinine was determined by its reaction with picric acid (Jaffe reaction), which forms a yellowish-red chromogen whose intensity, measured by spectrophotometry (λ = 510 nm), is proportional to its concentration (mg/dL) [47]. 

Urea

Urea concentration was measured in serum, based on the reaction between ammonia, formed by hydrolysis of urea in an aqueous medium, with alpha-ketoglutarate and NADH, catalyzed by glutamate dehydrogenase (GLDH), which forms glutamate and NAD+. NADH consumption, measured by spectrophotometry (λ = 340 nm), is proportional to urea concentration [48].

#### 4.6.5. Histopathological Analysis

The heart, liver, stomach, and kidneys were weighed, fixed in buffered formalin (10%), and embedded in paraffin. Sections of 5 μm (thick) were then obtained, which were subjected to an alcohol-xylene series and mounted on slides. Staining was performed with hematoxylin and eosin (H&E), and the examination was made under an optical microscope (Nikon Eclipse E200).

### 4.7. Antinociceptive Activity

#### 4.7.1. Acetic-Acid-Induced Peritonitis

Based on the Koster et al. (1959) [49] model, cavity inflammation was induced by ip injection of ACA (0.6% *v*/*v*) in mice (*n* = 7/group) pretreated (60 min) with saline added 4% DMSO (Control); MnE increasing doses (200, 400, and 800 mg/kg) or indomethacin (INDO, 10 mg/kg). Nociception was then measured by the number of writhes manifested between 10 to 30 min after ACA administration. 

After euthanasia by cervical dislocation under anesthesia, 3 mL of saline was injected into the peritoneal cavity of the animals. Peritoneal wash was collected, and protein concentration (mg/mL) was measured by Lory’s (1951) method [50], as an indirect marker of plasma leakage. A group that was not given ACA (white) was added to determine baseline protein concentrations.

#### 4.7.2. Formalin Test (FT)

To assess the participation of neural and/or inflammatory processes, biphasic nociception was induced by plantar injection (sc; right hind paw) of 20 μL of formalin (0.92%) [51] of mice pretreated (60 min) with saline added 4% DMSO (Control), MnE (400 mg/kg), or morphine (4 mg/kg). Morphine was administrated 30 min before the noxious stimulus. 

Nociception was measured through the time expended licking the formalin-injected paw, being evaluated in two phases: phase I (0–5 min), neuropathic nociception, which is triggered by direct stimulation of sensory terminals by formalin; and phase II (15–30 min) is inflammatory nociception, generated by the consequent inflammatory process [22].

#### 4.7.3. Open Field (OF) Test

To verify possible effects on consciousness or mobility that could compromise the animal’s performance submitted to formalin test, control and MnE groups were evaluated in the open field, as described above, 5 min before exposure to the noxious stimulus.

### 4.8. Statistical Analysis

Data are presented by the mean ± standard error of the mean (SEM). The Shapiro–Wilk method was applied to evaluate the data distribution. Difference between groups with Gaussian distribution was evaluated by Student’s *t*-test, one-way ANOVA, followed by Dunnett’s test, or one-way RM-ANOVA, followed by Holm Sidak’s test. Groups with a non-Gaussian distribution were evaluated using Mann–Whitney’s test; Kruskal–Wallis’s test followed by Dunn’s test or Friedman’s test. We considered statistically significant the differences with *p* < 0.05.

## 5. Conclusions

In summary, our results present the chromatographic profile of MnE (ethanolic extract of *M. nobilis*), identifying 44 secondary metabolites, classified as phenolic acids, flavonoids, and hydrolyzed tannins. We demonstrate that its LD_50_ for acute oral administration is above 2000 mg/kg, being classified as a low-toxicity xenobiotic. It also shows its activity in reducing nociception, as well as inhibiting plasma extravasation, both associated with inflammatory processes, which probably involves the ability of its secondary metabolites, such as ellagic acid, gallic acid, methyl gallate, *p*-coumaric acid, quercetin 3-O-xyloside, quercetin, and kaempferol, to reduce the synthesis and/or release of inflammatory enzymes and mediators, such as COX-2, iNOS, cytokines, prostanoids, and NO, in addition to increasing the production of anti-inflammatory cytokines and presenting antioxidant activity. We hypothesize, therefore, that the MnE antinociceptive and plasma leakage inhibition properties are the product of the synergistic action of its constituents, still considering that several of the substances annotated in the MnE still need to have their properties elucidated, as well as other mechanisms to be explored, which should compose future investigations. The present study, therefore, highlights the potential of this Amazonian species for the development of therapeutic agents devoted to the treatment of painful and inflammatory conditions.

## Figures and Tables

**Table 1:** Compounds identified by LC-HRMS-ToF in *Margaritaria nobilis* ethanolic extract (MnE).

Peak	RT (min)	Compound	Formula	[M−H]−Exp.	Error (ppm)
1	1.84	Gallic acid	C_7_H_6_O_5_	169.0138	0.6
2	4.51	*O*-Coumaroylgalactaric acid	C_15_H_16_O_10_	355.0661	1.1
3	4.91	*O*-Feruloylgalactaric acid	C_16_H_18_O_11_	385.0766	1.3
4	5.17	Methyl gallate	C_8_H_8_O_5_	183.0285	4.4
5	5.92	Galloyl-DHHDP-HHDP-glucose	C_41_H_28_O_27_	951.0703	3.9
6	6.09	Galloyl-HHDP-glucose	C_27_H_22_O_18_	633.0710	2.8
7	6.56	Galloyl-Che-HHDP-glucose Isomer I	C_41_H_30_O_27_	953.0888	0.8
8	6.56	Trigalloyl-glucose	C_27_H_24_O_18_	635.0866	2.8
9	6.92	*p*-Coumaric acid	C_9_H_8_O_3_	163.0389	3.7
10	6.98	Quercetin 3-*O*-glucosyl-glucoside	C_27_H_30_O_17_	625.1368	5.9
11	7.18	Ethyl gallate	C_9_H_10_O_5_	197.0445	2.5
12	7.24	Phyllanthusiin C Isomer	C_40_H_30_O_26_	925.0983	3.6
13	7.55	Ellagic acid *O*-xyloside	C_19_H_14_O_12_	433.0410	0.7
14	7.67	Quercetin 3-*O*-xylosyl-glucoside	C_26_H_28_O_16_	595.1321	3.7
15	7.84	Quercetin 3-*O*-rhamnosyl-glucoside	C_27_H_30_O_16_	609.1427	4.8
16	7.87	Ellagic acid *O*-rhamnoside	C_20_H_16_O_12_	447.0585	4.7
17	7.96	Galloyl-Che-HHDP-glucose Isomer II	C_41_H_30_O_27_	953.0904	0.8
18	8.01	Ellagic acid	C_14_H_6_O_8_	300.9972	4.0
19	8.39	Digalloyl-HHDP-glucose	C_34_H_26_O_22_	785.0847	1.3
20	8.39	Methyl neochebulagate Isomer	C_42_H_34_O_28_	985.1155	0.3
21	8.62	Quercetin 3-*O*-glucoside Isomer I	C_21_H_20_O_12_	463.0890	2.8
22	8.73	Excoecariphenol C Isomer	C_37_H_30_O_24_	857.1077	3.3
23	8.73	Tetragalloyl-glucose	C_34_H_28_O_22_	787.0977	2.2
24	8.76	Kaempferol 3-*O*-rhamnosyl-glucoside	C_27_H_30_O_15_	593.1528	3.7
25	8.87	Kaempferol 3-*O*-xylosyl-glucoside	C_26_H_28_O_15_	579.1376	4.0
26	8.87	Quercetin 3-*O*-glucoside Isomer II	C_21_H_20_O_12_	463.0898	4.5
27	8.93	Di-*O*-Methyl ellagic acid *O*-glucoside	C_22_H_20_O_13_	491.0852	5.3
28	9.41	Quercetin 3-*O*-rhamnosyl-xyloside	C_26_H_28_O_15_	579.1350	0.0
29	9.61	Quercetin 3-*O*-xyloside	C_20_H_18_O_11_	433.0765	1.4
30	9.70	Kaempferol 3-*O*-glucoside Isomer I	C_21_H_20_O_11_	447.0935	1.8
31	9.95	Galloyl-HHDP-di-deoxyglucose	C_27_H_24_O_16_	603.0945	6.8
32	10.15	Trigalloyl-dideoxyglucose	C_27_H_24_O_16_	603.1013	4.5
33	10.24	Kaempferol 3-*O*-glucoside Isomer II	C_21_H_20_O_11_	447.0914	2.9
34	10.61	Kaempferol 3-*O*-rhamnosyl-xyloside	C_26_H_27_O_14_	563.1431	5.3
35	10.69	Kaempferol 3-*O*-xyloside	C_20_H_18_O_10_	417.0836	3.4
36	10.78	Methylellagic acid *O*-rhamnoside	C_21_H_18_O_12_	461.0736	3.5
37	11.01	Phyllanthusiin A Isomer	C_41_H_28_O_27_	951.0743	0.3
38	12.10	Trigalloyl-HHDP-glucose	C_41_H_30_O_26_	937.0962	1.6
39	12.29	Phyllanthusiin U Isomer	C_40_H_28_O_26_	923.0801	1.1
40	14.00	Quercetin	C_15_H_10_O_7_	301.0334	4.7
41	14.91	Methylquercetin 3-*O*-glucoside	C_22_H_22_O_12_	477.1018	3.1
42	16.91	Kaempferol	C_15_H_10_O_6_	285.0399	0.0
43	18.14	Galloyl-Cinnamoyl-HHDP-glucose	C_36_H_28_O_19_	763.1154	0.9
44	19.04	Tri-*O*-methyl ellagic acid	C_17_H_12_O_8_	343.0450	1.2

**Table 2:** Effects of *Margaritaria nobilis* leaf’s ethanolic extract (MnE) acute oral administration (2000 mg/kg) on differential leukocyte count. Data presented as mean ± SEM (*n* = 4–5/group). Student’s *t*-test.

Leukocytes	Control (%)	MnE 2000 mg/kg (%)	*p*
Segmented neutrophils	37.50 ± 0.64	33.60 ± 2.09	0.153
Banded neutrophils	0.50 ± 0.29	0.20 ± 0.20	0.407
Lymphocytes	59.50 ± 1.19	64.40 ± 2.48	0.147
Monocytes	2.50 ± 0.65	1.80 ± 0.37	0.356
Basophils	0.00 ± 0.00	0.00 ± 0.00	1.000
Eosinophils	0.00 ± 0.00	0.00 ± 0.00	1.000

## Data Availability

Data is contained within the article and supplementary material.

## Supplementary Materials

The following supporting information can be downloaded at: https://www.mdpi.com/article/10.3390/ph16050689/s1, Figure S1: Histological evaluation of the stomach, kidney, liver, and heart of rats acutely treated with MnE limit dose.
