# Supplementary material for: Margaritaria nobilis L.f. (Phyllanthaceae) Ethanolic Extract: Low Acute Oral Toxicity and Antinociceptive Activity
Source: Pharmaceuticals (Basel). 2023 May 3;16(5):689. doi: 10.3390/ph16050689 (PMC10221426; doi:10.3390/ph16050689)

pharmaceuticals

Supplementary Material

for

Margaritaria nobilis L.f. (Phyllanthaceae) Ethanolic Extract: Low Acute Oral Toxicity and Antinociceptive Activity

Fabiana Menezes S. Camara [1], Brenda Costa da Conceição [1,2], Eloise Karoline S. Cardoso [1,2], Johan Carlos C. Santiago [3], Carlos Alberto B. Albuquerque [3], Washington L. Pereira [4], Marta C. Monteiro [2], Consuelo Y. Yoshioka e Silva [2,3], Milton Nascimento da Silva [2,3], Cristiane F. Maia [1,2] and Eneas A. Fontes-Junior [1,2,*]

[1] Laboratory of Inflammation and Behavioral Pharmacology (Lafico), Health Science Institute, Federal University of Pará, Belém 66075110, PA, Brazil
[2] Pharmaceutical Sciences Post-Graduation Program, Health Sciences Institute, Federal University of Pará, Belém 66075110, PA, Brazil
[3] Laboratory of Liquid Chromatography (Labcrol), Exact and Natural Sciences Institute, Federal University of Pará, Belém 66075110, PA, Brazil
[4] Animal Pathology Laboratory, Amazon Federal Rural University, Belém 66077830, PA, Brazil
* Correspondence: efontes@ufpa.br

Figure S1 | Histological evaluation of the stomach (**A** – Control and **B** – MnE; Barr - 200µm), kidney (**C** – Control and **D** – MnE; cortex; Barr - 100µm), liver (**E** – Control and **F** – MnE; Barr - 100µm), and heart (**G** – Control and **H** – MnE; Barr - 100µm) of rats acutely treated with MnE limit dose (2,000 mg/kg; orally). Hematoxylin and eosin staining. No changes indicative of toxicity was identified. All samples showed histological normality.

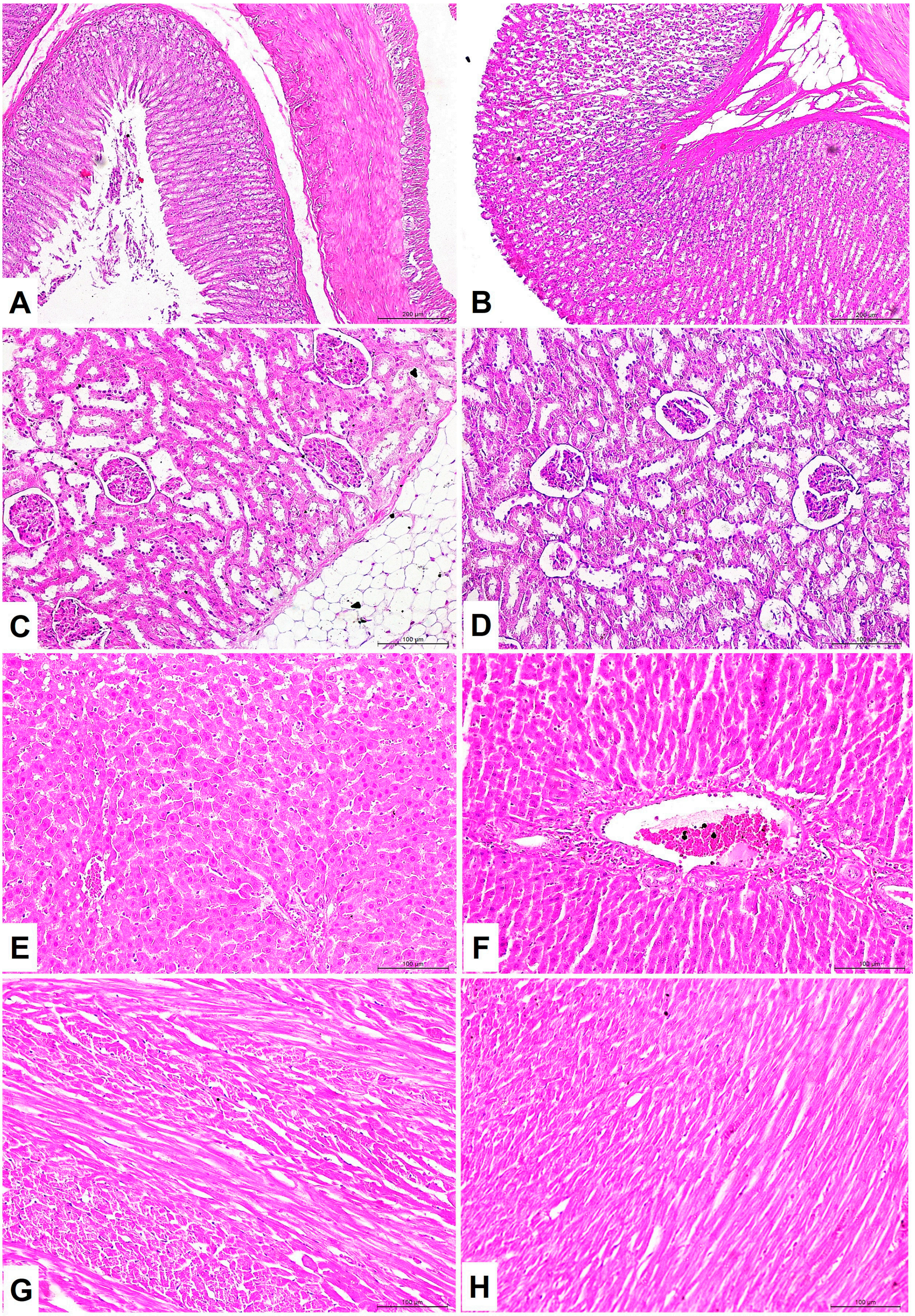

Supplement: Supplementary file 1 [file pharmaceuticals-16-00689-s001.zip › pharmaceuticals-2295963-supplementary.pdf]
